# Emphysematous Cystitis

**DOI:** 10.7759/cureus.11723

**Published:** 2020-11-27

**Authors:** Oluwadamilola A Adeyemi, John P Flaherty

**Affiliations:** 1 Department of Medicine, Section of Infectious Diseases, Swedish Hospital - NorthShore University HealthSystem, Chicago, USA; 2 Department of Medicine, Section of infectious Diseases, Northwestern Medicine Lake Forest Hospital, Lake Forest, USA; 3 Department of Medicine, Division of Infectious Diseases, Northwestern University Feinberg School of Medicine, Chicago, USA

**Keywords:** emphysematous cystitis, urinary tract infection, complicated urinary tract infection

## Abstract

Emphysematous cystitis is a relatively rare and potentially life-threatening condition characterized by the collection of gas in the bladder wall and lumen due to infection caused by gas-forming organisms. Imaging studies are necessary to detect emphysematous cystitis. The management consists of broad-spectrum antibiotics, strict glycemic control, and bladder drainage. Complications may arise in some cases, requiring surgical treatment. We present a case of extended spectrum beta-lactamase producing *Escherichia coli *and *Klebsiella pneumoniae* emphysematous cystitis in a known diabetic.

## Introduction

Emphysematous cystitis, also known as cystitis emphysematosa, is a condition in which pockets of gas are formed within the bladder wall and lumen due to infection caused by gas-forming organisms (*Escherichia coli, Klebsiella pneumoniae, Enterobacter aerogenes, Proteus mirabilis, Streptococcus *species,* *or *Clostridium perfringens*) [1-5]. It is commonly seen in elderly diabetic women [3, 5]. The median age of patients at the presentation in a review was 66 years [2]. Cases are reported twice as often in women as in men [1, 3]. Other risk factors include glycosuria, neurogenic bladder, recurrent urinary tract infection (UTI), and urinary stasis secondary to bladder outlet obstruction (BOO) [1-3]. Alcoholism, undernourishment, and disabled general medical conditions have also been associated in some reports [4, 5]. Among these risk factors, diabetes mellitus (DM) appears to be the strongest, as seen in 50% to 70% of cases [1-3]. It is considered to be a form of complicated UTI [2-5].

## Case presentation

A 76 year-old-man with DM controlled on metformin was admitted for a decline in his overall well-being. His friends reported he had become fatigued, forgetful, less involved, missed medications, and had lost some weight. He reported passing foul-smelling dark urine but denied dysuria, frequency, fevers, chills, or rigors. He denied abdominal pain, nausea, vomiting, or a history of recent urinary bladder instrumentation. 

**Figure 1:**
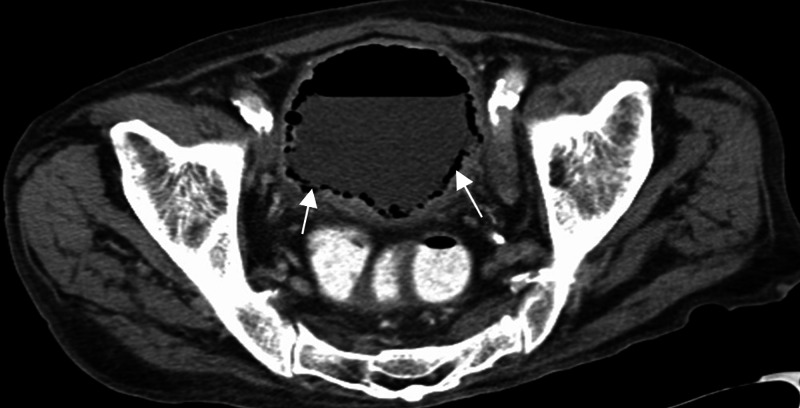
CT abdomen and pelvis axial section through the urinary bladder Diffuse bladder wall thickening with air seen circumferentially within the bladder wall (arrows).

**Figure 2:**
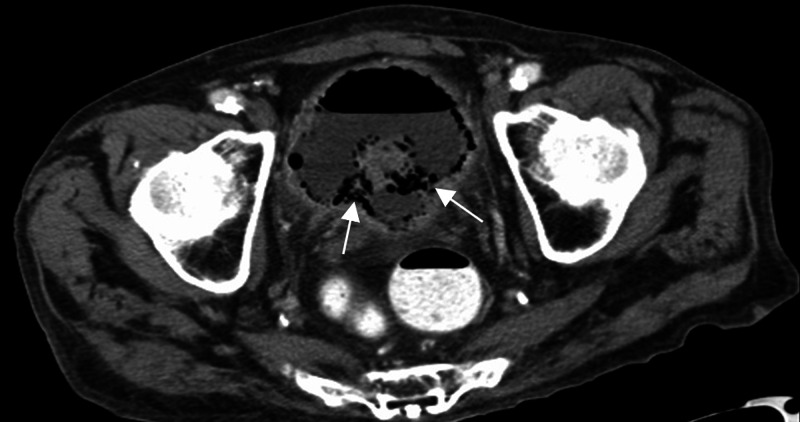
CT abdomen and pelvis axial section through the urinary bladder Diffuse bladder wall thickening with air seen circumferentially within the bladder wall (arrows).

On admission, his oral temperature was 98°F, blood pressure was 166/94 mmHg, pulse was 66 beats per minute and the respiratory rate was 18 breaths per minute. His physical examination was unremarkable, and he was alert and oriented to person, place and year. Complete blood count was normal, but serum creatinine was 1.7 mg/dl. Other electrolytes, including sodium, were normal. His glycosylated hemoglobin was 5.6%. Computed tomography (CT) of the brain showed moderate to severe small vessel ischemic changes but no acute abnormality. Rapid plasma reagin (RPR) was negative, and vitamin B12 and thyroid-stimulating hormone levels were normal. Urinalysis revealed pyuria and urine culture grew > 100,000 CFU/mL *Escherichia coli *and *Klebsiella pneumoniae*. Blood cultures obtained on admission were negative. CT images of the chest, abdomen, and pelvis performed to rule out malignancy showed diffuse bladder wall thickening with air seen circumferentially within the bladder wall (Figures 1-2).

Based on his clinical presentation, microbiologic and radiologic findings, he was diagnosed with emphysematous cystitis, a rare complication of lower urinary tract infection. He was initially treated with empiric intravenous piperacillin/tazobactam then discharged to a nursing facility on ertapenem (*Escherichia coli* strain was an extended spectrum beta-lactamase producer). A urinary catheter was inserted for effective bladder drainage, and he completed a total of 14 days of antibiotic therapy. After completion of antibiotic therapy, a repeat CT of the abdomen and pelvis showed complete resolution of the bladder wall emphysematous changes.

## Discussion

The clinical presentation of emphysematous cystitis ranges from being asymptomatic to pneumaturia or irritative voiding symptoms to acute abdomen with severe sepsis [2, 3]. In asymptomatic patients (which occurs in up to 7% of cases), it may be incidentally diagnosed when imaging is performed for other reasons [1-4]. Amongst symptomatic patients, abdominal pain is seen in 80% of patients, gross hematuria in 60% of patients, fever may be observed in approximately 30% to 50% of patients, and ischuria is seen in 10% of patients [3]. Frank peritoneal signs were seen in 6.2% of patients [5]. Although pneumaturia appears to be a highly specific symptom, it is a rare patient complaint [3]. Pneumaturia is observed in 70% of patients with bladder catheterization. Other acute cystitis symptoms (dysuria, urinary frequency, and urinary urgency) occur in approximately 50% of patients [3, 5]. However, these symptoms are nonspecific and usually mild, if they exist. For this reason, no significant clinical features strongly suggestive of emphysematous cystitis have been reported [3]. The exact mechanism contributing to the formation of gas is unknown [2, 5]. Various theories have been suggested, including fermentation of glucose or albumin by microorganisms infecting the bladder, emphasizing disequilibrium between gas formation and clearance [1, 2, 5]. Although our patient had well-controlled DM based on his glycosylated hemoglobin level, he had glycosuria on admission. Of note, diabetics with no glycosuria, patients with well-controlled DM, and nondiabetic patients also develop emphysematous infections of the urinary tract [2, 5]. *Escherichia coli* and *Klebsiella pneumoniae *are often isolated from urine cultures [1-3]. In the review of 135 cases by Thomas and colleagues, *Escherichia coli* was the causative pathogen in 58% of cases, followed by *Klebsiella pneumoniae *in 21% [2]. Other pathogens reported in that review were* Enterobacter aerogenes, Clostridium perfringens, Clostridium welchii, Proteus mirabilis, Pseudomonas aeruginosa, Group D Streptococcus, Enterococcus faecalis, Staphylococcus aureus, Candida albicans, Candida tropicalis*, and *Aspergillus fumigatus* [2]. These species can ferment glucose and lactate to yield various gases, such as nitrogen, hydrogen, oxygen, and carbon dioxide, that collect in the submucosa or lumen of the bladder [1, 3]. 

Performing blood cultures, urinalysis, Gram staining of the urine and urine cultures are essential for detecting the responsible pathogen and helps to select the appropriate antibiotic regimen. The presence of hematuria and pyuria with positive urine cultures is usually revealed in this process. About half of the patients are reported to have bacteremia [3]. Selected antibiotics should be tailored to the culture and sensitivity report [2]. Since carbon dioxide is readily absorbed in human tissue, eventual resolution usually occurs after eliminating the infecting microorganism. Because of the spectrum of illness produced by this disease, a systemic approach to diagnosis and management is not feasible. Evaluation of the emphysematous process must be tailored to the individual patient [1]. A delayed diagnosis can result in bladder rupture, overwhelming infection, and death [3]. A complicated course is seen in 19% of cases, and the overall reported mortality rate ranged from 3% to 12% [2, 3, 5]. However, when emphysematous cystitis occurs in concert with another gas-forming disease within the upper urinary tract, emphysematous pyelonephritis, the mortality rate increases by up to 14% to 20% [3]. Successful management depends on early diagnosis with correction of any predisposing factor, effective glycemic control when required, prompt administration of appropriate antibiotics, adequate bladder drainage, and surgical excision of involved tissue when required [1-3]. Approximately 90% of cases are treated with medical therapy alone, whereas 10% require combined surgical and medical intervention. Surgery involved cystectomy, partial cystectomy, cystotomy, bladder lavage, or nephrectomy for combined cases with emphysematous pyelonephritis [2, 5].

Imaging methods, such as plain conventional abdominal radiography and CT, are pivotal for obtaining a definitive diagnosis [3]. Eighty-four percent of patients in the review of 135 cases were diagnosed using plain films of the abdomen, 40% were diagnosed using CT while 7% were diagnosed using bladder ultrasonography. More recently, CT was the primary imaging method [2]. The characteristic radiographic feature involves curvilinear areas of increased radiolucency delineating the bladder wall, separated from the more posterior rectal gas [2, 3]. The occurrence of intraluminal gas can be detected as an air-fluid level that is not static [3]. There often is a radiolucent line of gas around the bladder wall and gas within the bladder, giving a cobblestoned or beaded necklace appearance [1, 3]. Excretory urography will demonstrate gas within the bladder and can exclude coincident lesions in the renal tract [1]. Every diabetic patient with a urinary tract infection who seems to be severely ill should have an abdominal X-ray as a minimal screening tool to detect emphysematous complications [4]. Findings on X-ray may be confused with rectal gas, emphysematous vaginitis, and gas gangrene of the uterus [5]. Abdominal radiography and cystography may appear ominous, even during a benign course [1].

CT is the most sensitive and specific diagnostic tool [5]. It is commonly used and is necessary for diagnosis [3]. CT can clarify the extent and location of the gas collection [1]. CT also reveals the severity of the condition (e.g., ascending emphysematous infections, intra-abdominal abscesses, or adjacent neoplastic disease). Furthermore, other sources of pelvic air can be detected, such as trauma, fistulous connections with the bowel (vesicocolic fistula) or fistulous connection to the vaginal (vesicovaginal fistula), pneumatosis cystoides intestinalis, gas gangrene of the uterus, and vaginitis emphysematosa [3]. Ultrasound shows diffuse wall thickening and focally high-echoic regions with dirty acoustic shadowing [1, 3]. A cystoscopic examination is not essential for diagnosis, but it can add information about BOO, which can contribute to the development of emphysematous cystitis. Cystoscopy was used in 39% of cases, but the diagnosis was also confirmed radiologically in each case. Cystoscopy with CT can help to diagnose enterovesical fistula as a potential cause [2]. There is no specific information regarding the use of magnetic resonance imaging (MRI) for the diagnosis of emphysematous cystitis. The value of MRI seems limited because gas-forming infections can cause signal voids, which are difficult to interpret on MRI [5]. 

## Conclusions

Emphysematous cystitis is a rare complication of urinary tract infection due to gas-forming organisms that is potentially life-threatening. Diagnosis can be made using a plain abdominal radiograph; however, CT is considered to be the imaging modality of choice. Successful management depends on early diagnosis with correction of any predisposing factor, prompt administration of appropriate antibiotics, adequate bladder drainage, and surgical excision of involved tissue when required.
